# Retraction: Study of aerobic and anaerobic bacterial profile of nosocomial infections and their antibiotic resistance in a referral center, Southwest Iran: A three year cross-sectional study

**DOI:** 10.1371/journal.pone.0322159

**Published:** 2025-04-03

**Authors:** The PLOS One Editors

The *PLOS One* Editors retract this article [1] due to concerns about compliance with the PLOS Human Subjects Research policy. Ethics approval documentation provided in post-publication discussion was not sufficient to confirm compliance with this policy. The editors regret that this issue was not identified prior to publication.

ADK, AAZD, MH, MS, and FJM did not agree with the retraction. NA and FI either did not respond directly or could not be reached.

## References

[1] Ahmadkhosravi N, Khosravi AD, Asareh Zadegan Dezfuli A, Hashemzadeh M, Saki M, Mehr FJ, et al. Study of aerobic and anaerobic bacterial profile of nosocomial infections and their antibiotic resistance in a referral center, Southwest Iran: a three year cross-sectional study. PLoS One. 2021;16(11): e0259512. doi: 10.1371/journal.pone.0259512 34752474 PMC8577755

